# The Regenerative Potential of bFGF in Dental Pulp Repair and Regeneration

**DOI:** 10.3389/fphar.2021.680209

**Published:** 2021-07-20

**Authors:** Keyue Liu, Sijing Yu, Ling Ye, Bo Gao

**Affiliations:** State Key Laboratory of Oral Diseases, National Clinical Research Center for Oral Diseases, West China Hospital of Stomatology, Sichuan University, Chengdu, China

**Keywords:** fibroblast growth factors, basic fibroblast growth factor, regenerative endodontic therapy, regenerative endodontics, pulp repair, pulp regeneration

## Abstract

Regenerative endodontic therapy intends to induce the host’s natural wound-healing process, which can restore the vitality, immunity, and sensitivity of the inflammatory or necrotic pulp tissue destroyed by infection or trauma. Myriads of growth factors are critical in the processes of pulp repair and regeneration. Among the key regulatory factors are the fibroblast growth factors, which have turned out to be the master regulators of both organogenesis and tissue homeostasis. Fibroblast growth factors, a family composed of 22 polypeptides, have been used in tissue repair and regeneration settings, in conditions as diverse as burns, ulcers, bone-related diseases, and spinal cord injuries. Meanwhile, in dentistry, the basic fibroblast growth factor is the most frequently investigated. Thereby, the aim of this review is 2-fold: 1) foremost, to explore the underlying mechanisms of the bFGF in dental pulp repair and regeneration and 2) in addition, to shed light on the potential therapeutic strategies of the bFGF in dental pulp–related clinical applications.

## Introduction

With the advancement of our knowledge in pulp biology, the concept of treatment of endodontic diseases changes accordingly. “Regenerative endodontics” has been defined as “biologically based procedure designed to replace damaged structures, including dentin and root structures, as well as cells of the pulp-dentin complex” (Murray et al., 2007). The new era of regenerative endodontic therapy intends to induce the host’s natural wound-healing process, which can restore the vitality, immunity, and sensitivity of the inflammatory or necrotic pulp tissue destroyed by infection or trauma (Yang et al., 2016).

**FIGURE 1:**
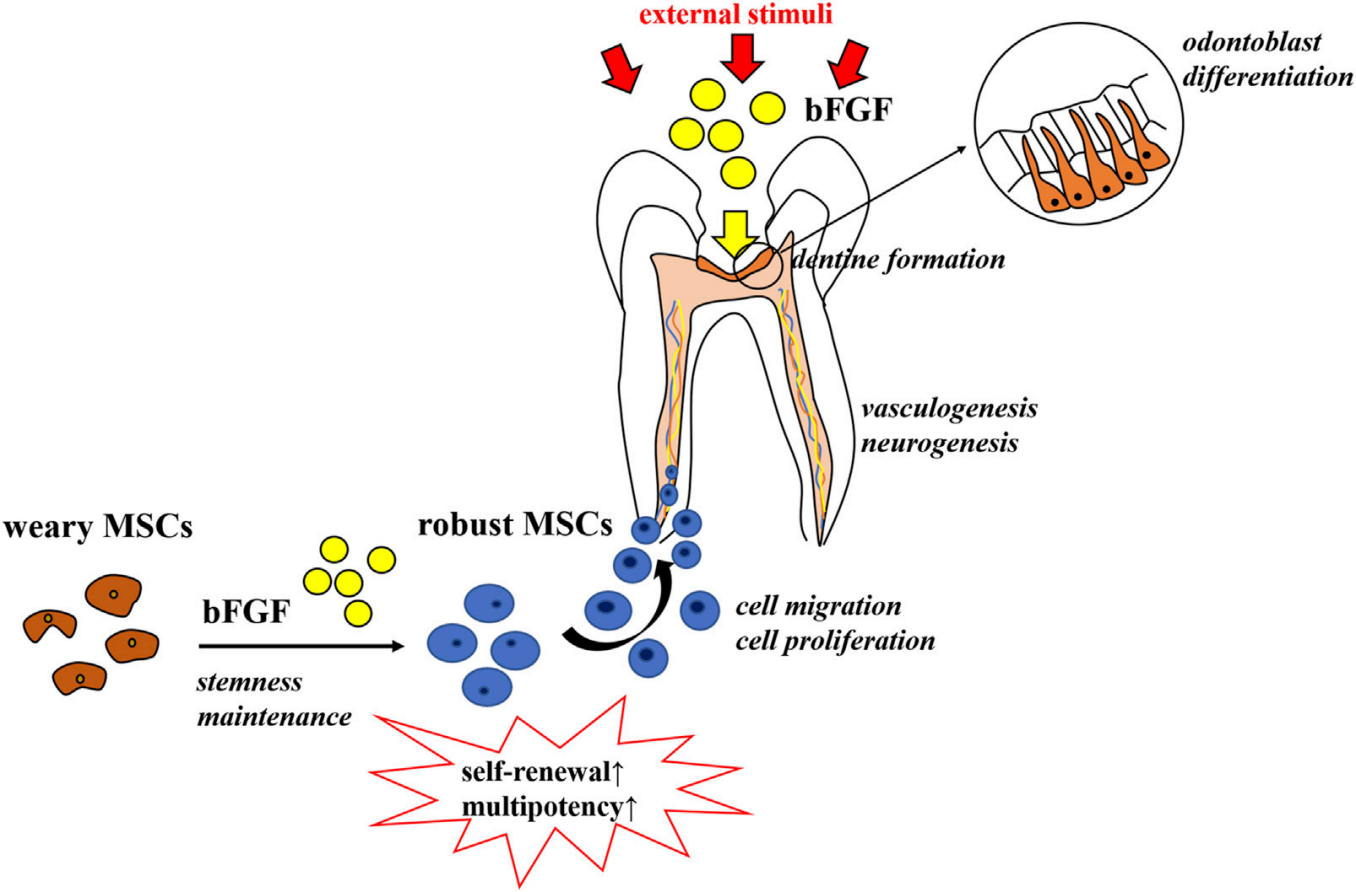
Role of the bFGF in pulp repair and regeneration. The bFGF contributes to the regulation of virtually all aspects of tooth development, repair, and regeneration, including mesenchymal stem cells’ stemness maintenance, migration, and proliferation, as well as dentine formation, vasculogenesis, and neurogenesis.

Both pulp repair and regeneration processes are orchestrated by a highly coordinated interplay of different growth factors and cytokines (Orti et al., 2018). These myriads of growth factors create a favorable microenvironment conducive to tissue repair and/or regeneration taking place and act as signaling molecules that regulate cell behaviors, including migration, proliferation, and differentiation (Kim et al., 2018). Among the key regulatory factors are the fibroblast growth factors (FGFs), which have turned out to be the master regulators of both organogenesis and tissue homeostasis (Maddaluno et al., 2017). FGFs are a family of growth factors, consisting of 22 members that share 13–71% sequence homology in mammals (Ornitz and Itoh, 2001). Major attention has been paid to the use of FGFs in tissue repair and regeneration settings, in conditions as diverse as burns, ulcers, bone-related diseases, and spinal cord injuries, just to name a few (EL Agha et al., 2016; Nunes et al., 2016; Zhou et al., 2018; Wang J. et al., 2019). Meanwhile, in dentistry, the basic fibroblast growth factor (bFGF), also termed FGF-2, is the most frequently investigated (Vaseenon et al., 2020). As a member of the FGF family, the bFGF has been implicated as a signaling molecule that contributes to the regulation of virtually all aspects of tooth development, repair, and regeneration (Li et al., 2014; Vaseenon et al., 2020), including mesenchymal stem cell migration, proliferation, and stemness maintenance, as well as dentine formation, vasculogenesis, and neurogenesis (as shown in Figure 1) (Galler et al., 2012; Chang et al., 2017).

To figure out the role of the bFGF in regenerative endodontics, this review aims to give a comprehensive summary of the underlying mechanisms associated with the bFGF in promoting dental pulp repair and regeneration and shed light on the potential therapeutic strategies of the bFGF in dental pulp–related clinical applications.

## The Underlying Mechanisms of Basic Fibroblast Growth Factor in Dental Pulp Repair and Regeneration

According to the mode of action, the bFGF acts through binding to tyrosine kinase FGF receptors 1–4 (FGFR1–4) on the cell membrane (Müller et al., 2012). When the bFGF binds to its receptors, phosphorylation occurs and subsequently triggers a cascade of intracellular signaling pathways, such as the RAS-MAPK, PI3K-AKT, PLCγ, and JAK/STAT signaling pathways, initiating cell migration, proliferation, and differentiation (Müller et al., 2012). These findings lead to the hypothesis that the bFGF may use different intracellular signaling pathways to control specific biological processes in dental pulp repair and regeneration (Nowwarote et al., 2020). In this regard, we try to elucidate the potential roles of the bFGF in the specific biological processes, respectively.

### Cell Migration

Before the regeneration occurs, cells must migrate to the sites where they are required to function (Adam and Richard, 1999). The migration process is initiated by more than 50 chemotactic factors which have been identified in mammals (Horuk, 2001). Among those chemotactic factors, the bFGF has been reported to induce the migration of certain types of mesenchymal stem cells (MSCs) which are critical for pulp regeneration, including adult dental pulp stem cells (DPSCs), stem cells from human exfoliated deciduous teeth (SHEDs), stem cells from apical papilla (SCAPs), bone marrow–derived mesenchymal stem cells (BMMSCs), and so on (Peng et al., 2009). Several cytokines were examined to enhance the migratory ability of BMMSCs, while the bFGF in particular was able to initiate the migration of BMMSCs in a dose-dependent manner *via* the Akt/protein kinase B (PKB) pathway (Schmidt et al., 2006). It was reported that low concentrations of the bFGF attracted BMMSCs, whereas higher concentrations resulted in an ambivalent effect (Schmidt et al., 2006). The ability of the bFGF to induce the migration of DPSCs was also verified (Howard et al., 2010), which agreed with the later study that reported that the bFGF significantly recruited more DPSCs seeded on the surface of 3D collagen gel cylinders into the deep (Suzuki et al., 2011). Coincidentally, Fayazi et al. claimed that the number of SCAPs recruited by the G-CSF and bFGF was approximately 2-fold greater than that of other tested factors (Fayazi et al., 2017), and there was no apparent difference between the G-CSF and the bFGF in homing effect (Takeuchi et al., 2015). The periodontal ligament was also reported as a good source of MSCs (PDLSCs), which have a similar feature to that of BMMSCs and DPSCs, and could also contribute to the regenerative dentistry (Trubiani et al., 2008). In this regard, Zhang et al. verified that the bFGF promoted PDLSC migration and adhesion with more prominent ability than the VEGF (Zhang et al., 2013). Furthermore, the implantation of collagen scaffolds containing the bFGF resulted in abundant cell ingrowth, recellularization, and revascularization in endodontically treated root canals (Kim J. Y. et al., 2010).

Collectively, the bFGF is an efficacious chemotactic factor to facilitate the motility of MSCs and the following tissue remodeling processes, thereby providing a therapeutic basis for the cell homing strategy (Kim J. Y. et al., 2010; Galler et al., 2014).

### Cell Proliferation

Generally, stem cell populations of various oral cells would dramatically decrease during the culture period (Min et al., 2011), which greatly limited their applications in tissue regeneration.

The mitogenic ability of the bFGF has attracted much attention. Morito et al. showed that the ratio of dental pulp cells in the S-phase and the ratio of hDPCs expressing STRO-1 were significantly higher when in the presence of the bFGF (Morito et al., 2009). The mitogenic effect of the bFGF on dental pulp cells was also supported recently by adding it in culture medium. Promising results were received in that the bFGF significantly promoted the proliferation of DPSCs through activating the ERK pathway without comprising cell stemness and pluripotency (Luo et al., 2021). With no exceptions, the mitogenic potential of the bFGF on SHEDs was also found (Sukarawan et al., 2014), and further study revealed that the bFGF promoted SHED colony-forming units *via* the FGFR/PI3K pathway (Nowwarote et al., 2020). In the gene level, the bFGF was reported to be in charge of controlling the cell cycle progression by significantly increasing the MKI67 mRNA and Ki67 protein level in SHEDs (Nowwarote et al., 2020) and regulating the cdc2 and cyclin B1 expression in HDPCs and SCAPs in a dose-dependent manner *via* the MEK/ERK pathway (Chang et al., 2017; Chang et al., 2020), leading to a higher G2/M subpopulation and promoting the cell proliferation process (Nowwarote et al., 2020).

Taking those pieces of evidence together, the bFGF has the superiority to promote cell proliferation and control the cell cycle progression *via* stimulating the expression of related genes and proteins, including Ki67, cdc2, and cyclin B1. Therefore, its mitogenic effect can be put into use to amplify the cell number in order to obtain the sufficient amount of stem cells required for endodontic regenerative treatments and can also brighten the future of stem cell banking and tissue engineering (Luo et al., 2021).

### Stemness Maintenance

Stemness maintenance has emerged to preserve the two defined features of stem cells, self-renewal and multipotency, which would become limited when these cells are introduced in long-term culture (Kong et al., 2019). Interestingly, the bFGF added in growth medium was proven to be able to support the self-renewal of human embryonic stem cells and maintain them in a multipotent state (Ehman et al., 2006; Diecke et al., 2008). In addition, the bFGF has also been demonstrated to maintain MSC potential in multilineage differentiation. Correspondingly, the bFGF, in low-density cultures, was reported to retain the osteogenic, adipogenic (Tsutsumi et al., 2001), and chondrogenic potential (Solchaga et al., 2005) of BMMSCs through the regulation of the MAPK and Wnt signaling pathways (Solchaga et al., 2005). Although the expression of mesenchymal stem cell markers was generally decreased during culture, treatment with the bFGF could delay their decrements and maintain their expressions in oral stem cells during culture (Lee et al., 2015). Additional studies demonstrated that the bFGF regulated stemness maintenance by increasing the gene expression of pluripotent stem cell markers including NANOG, OCT4, and REX1 on SCAPs, DPSCs, and SHEDs (Wu et al., 2012; Osathanon et al., 2011; Sukarawan et al., 2014). It was further revealed that the bFGF induced REX1 expression *via* the FGFR/Akt pathway with IL-6 as a middle regulator (Nowwarote et al., 2017).

Taken together, these investigations imply the important role of the bFGF in maintaining human stem cells’ stemness, which is a critical step toward the clinical application of MSCs in regenerative endodontics.

### Dentinogenesis

Once subjected to external injuries, the pulp cells may go through proliferation and differentiation, developing into odontoblast-like cells to elicit reparative dentinogenesis, which is critical for the pulpal wound-healing process (Yamamura, 1985). Dentinogenesis is a complex and multistep process, which is regulated by various growth factors including the bFGF (Cooper et al., 2010). However, the effects of the bFGF on mineralization and odontoblast differentiation remain elusive, while both stimulatory and inhibitory effects of the bFGF on dentinogenesis have been reported. Several studies have shown that the bFGF inhibited dentinogenesis and the expression of dentin sialophosphoprotein (Dspp) and alkaline phosphatase (ALP) (Tsuboi et al., 2003; Takedachi et al., 2009; Kim et al., 2014). On the other hand, other studies have shown that the bFGF stimulated the formation of osteodentin and the expression of Dspp and ALP (Kikuchi et al., 2007; Ishimatsu et al., 2009; Kim Y.-S. et al., 2010). Thus, to gain better insight into the biphasic role of the bFGF in dentinogenesis and cell differentiation in the odontoblast lineage, the underlying mechanisms of these controversial results need to be elaborated upon.

Dental pulp cells at different stages of odontoblast differentiation were identified by using a series of transgenic mice (Sagomonyants and Mina, 2014a). Studies have shown that 2.3-GFP and 3.6-GFP transgenes were activated at early stages of odontoblast differentiation (polarizing odontoblasts), and DMP1-GFP first emerged in functional/secretory odontoblasts. Meanwhile, all three transgenes (2.3-GFP, 3.6-GFP, and DMP1-GFP) were expressed at high levels in fully differentiated/mature odontoblasts (Braut et al., 2003; Balic et al., 2010; Balic and Mina, 2011). In addition, researchers have generated a new kind of transgenic mouse using the bacterial artificial chromosome (BAC), directing the expression of the DSPP-Cerulean transgene that can be used to identify fully differentiated odontoblasts in the heterogeneous pulp cultures (Sagomonyants and Mina, 2014a). Cells were isolated and first grown for 7 days in medium supporting their proliferation (proliferation phase) and then for an additional 7 days in medium inducing their mineralization (differentiation/mineralization phase) (Sagomonyants and Mina, 2014b). Experiments conducted showed that the effects of the bFGF on odontogenic differentiation of pulp cells were stage-specific and depended on the stage of maturity of cells (Sagomonyants and Mina, 2014a). During the proliferation phase of *in vitro* growth, early and limited exposure to the bFGF increased the expression of the markers of dentinogenesis and the percentage of DMP1-GFP + functional odontoblasts, showing the differentiation of early progenitors into functional odontoblasts. During the mineralization/differentiation phase, additional/continuous exposure to the bFGF decreased the expression of the markers of dentinogenesis and the expression of DMP1-GFP and DSPP-Cerulean transgenes, indicating the shrinkage of the extent of mineralization and the failure of mature odontoblast differentiation. However, immediate withdrawal of bFGF for 7 days rapidly and mostly completed the recovery of mineralization and the expression of various GFP transgenes and dentinogenic markers (Sagomonyants and Mina, 2014a). Collectively, these results suggested a positive role of the bFGF in early differentiation into functional odontoblasts but a negative role in further differentiation into fully differentiated/mature odontoblasts.

Additional experiments were in progress to examine the underlying mechanisms mediating the biphasic effects of the bFGF on odontogenic differentiation. Studies revealed that the effects of the bFGF on odontoblast differentiation were mediated through the activation of FGFR/MEK/Erk1/2 signaling (Sagomonyants et al., 2015; Sagomonyants et al., 2017), and additional research examined that the BMP and Wnt signaling pathways also participated in the process (Sagomonyants and Mina, 2014b). Early and limited bFGF treatment promoting odontogenic differentiation during the proliferation phase was associated with increased levels of the components of the BMP (Bmp2, Dlx5, Msx2, and Osx) and Wnt (Wnt10a and Wisp2) pathways and with a decreased expression of the Wnt signaling inhibitor (Nkd2). Further addition of the bFGF suppressing the terminal odontogenic differentiation during the differentiation/mineralization phase was accompanied by decreased expression of the components of the BMP signaling (Bmp2, Runx2, and Osx) and lower levels of Wnt inhibitors (Nkd2 and Dkk3) but increased expression of the Wnt signaling (Wnt10a). Taken together, these observations suggested that the BMP signaling could act as a positive regulator during odontoblast differentiation, while Wnt signaling stimulated early odontoblast differentiation but inhibited the terminal odontoblast differentiation (Sagomonyants et al., 2017). Furthermore, Vining et al. also revealed the possible mechanism of the early and limited exposure to the bFGF promoting odontogenic differentiation by expanding αSMA-tdTomato^+^ cells and accelerating their differentiation into odontoblasts (Vining et al., 2018). IFT80 (intraflagellar transport protein 80) may also be involved in the positive differentiation process by maintaining cilia formation and FGFR1 expression and subsequently activating the AKT, Hh, and BMP2 signaling pathways to drive DPSCs differentiating into the odontogenic lineage (Yuan et al., 2019a). Nevertheless, the attenuation of ITGA11, CTGF, and ATF4 levels (Novais et al., 2019) and the downregulation of the Pi/PPi ratio (Nowwarote et al., 2018) in bFGF-treated SHEDs may participate in the negative regulatory mechanisms of the bFGF in odontogenic differentiation.

**FIGURE 2:**
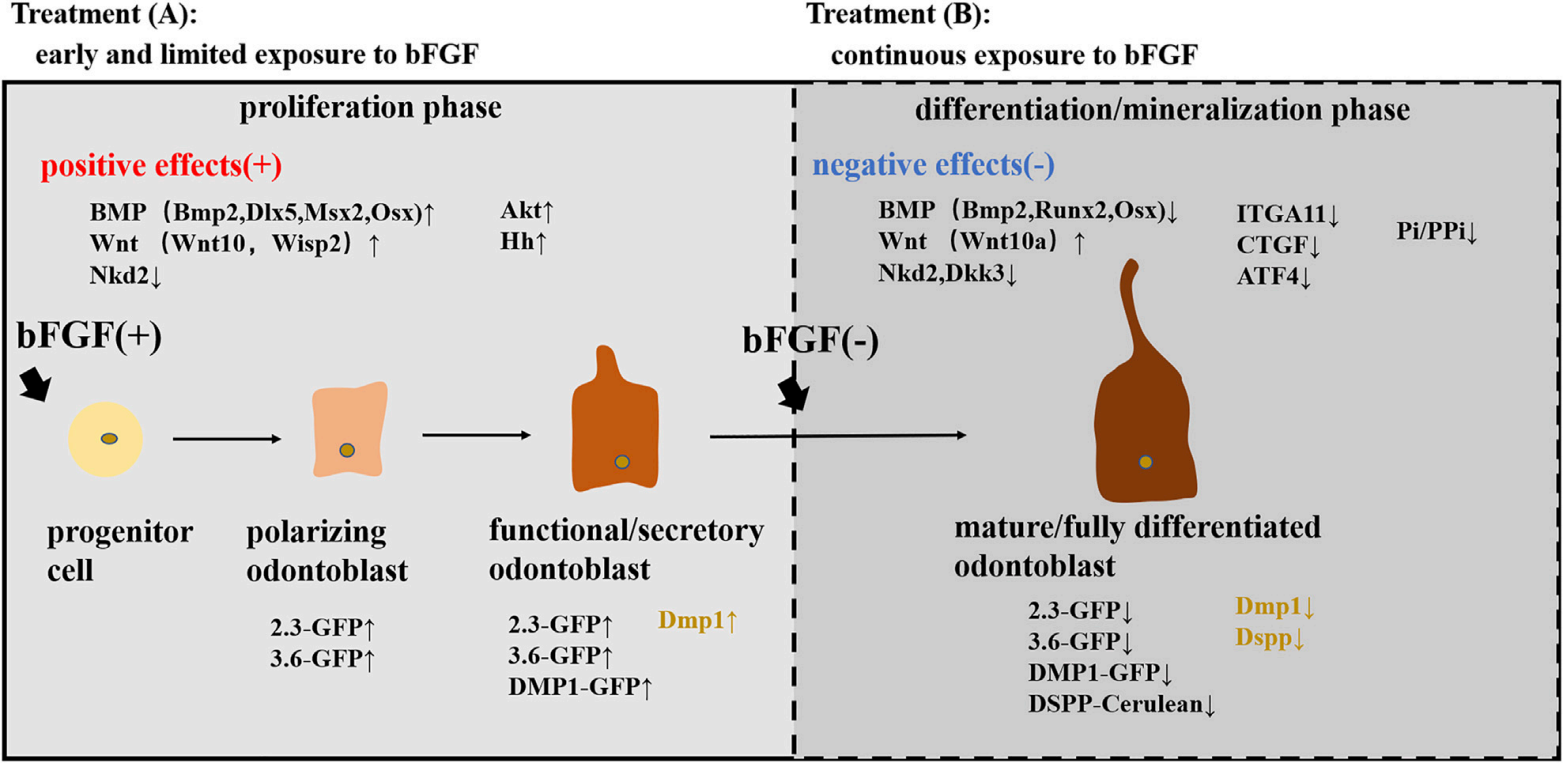
Stage-specific effects of the bFGF on odontoblast differentiation. Treatment **(A)**: early and limited exposure to the bFGF during the proliferation phase stimulated the differentiation into functional/secretory odontoblasts and increased the expression of various GFP transgenes (2.3-GFP, 3.6-GFP, and DMP1-GFP) and dentinogenic markers (Dmp1) by activating the BMP, Wnt, and Akt pathways. Treatment **(B)**: continuous exposure to the bFGF during the differentiation/mineralization phase inhibited the differentiation into mature odontoblasts and decreased the expression of GFP transgenes (2.3-GFP, 3.6-GFP, and DSPP-Cerulean) and dentinogenic markers (Dmp1 and Dspp) by activating the Wnt pathway while suppressing the BMP pathway and downregulating ITGA11, CTGF, and ATF4 levels and the Pi/PPi ratio.

These observations together show the stage-specific effects of the bFGF on dentinogenesis through dental pulp cells (as shown in Figure 2) and provide a better insight into the treatments for vital pulp therapy and dentinal regeneration by priming the dental pulp tissue with the bFGF to achieve the desired mineralization state, which may be able to impede the excessive calcification in the pulp (Qian et al., 2015).

### Neurogenesis

Neural regeneration is critical for ideal regenerative endodontic treatment so as to regain the normal sensation of the affected teeth (Kim et al., 2018). The bFGF is an important neurotrophin which possesses superior properties to promote neural stem cell (NSC) migration, proliferation, and self-renewal (Yeoh and de Haan, 2007). Ye et al. further informed that the bFGF could promote the survival and differentiation of NSCs to reduce brain damage and restore sensorimotor function after neonatal hypoxia-ischemia (Ye et al., 2018). The bFGF has also been reported to be able to maintain the survival of neuronal and glial cells, probably by protecting neurons from ROS-induced cell death and antagonizing the neuronal apoptosis induced by glutamate (Liu and Zhu, 1999). Indirectly, bFGF priming could protect cultured DPCs from hydrogen peroxide–induced cell death and increased the number of DPCs surviving so as to provide neurotrophic factors, thereby promoting axonal regeneration and locomotor function recovery (Nagashima et al., 2017).

DPSCs, deriving from the neural crest, retain remarkable characteristics that are similar to those of neural cells and have the potential to undergo neural differentiation to help crushed nerves achieve functional recovery and anatomical repair *in vivo* (Wang et al., 2020). The neurotrophic and neuroprotective merits of DPSCs make it an ideal stem cell source for neural repair and regeneration (Luo et al., 2018b; Wang D. et al., 2019). Accordingly, the implanted DPSCs could promote endogenous neural stem/progenitor cell proliferation, recruitment, and maturation through modulating the local microenvironment *via* secreting multiple factors, especially the bFGF (Huang et al., 2008). On the other hand, numerous protocols have documented that the exogeneous application of the bFGF could facilitate DPSCs differentiating into neurons. Studies discovered that the supplementation of the bFGF increased the neurosphere size and the neurogenic markers of DPSCs (Osathanon et al., 2011; Kang Y.-H. et al., 2019) and simultaneously revealed that the bFGF induced neuronal differentiation of DPSCs through the PLCγ signaling pathway (Osathanon et al., 2011). Meanwhile, Zheng et al. recognised the phospho-ERK (*p*-ERK) activation as a major mediator in such a process (Zheng et al., 2020). In addition, the bFGF and NGF were shown to have a synergistic effect to increase neural differentiation of DPSCs by upregulating the levels of Sirt1 and activating the ERK and Akt signaling pathways (Zhang et al., 2017). Coincidentally, the bFGF and NGF–co-transfected BMMSCs were also inclined to differentiate into neurons with the manipulation of the ERK and Akt signaling pathways (Hu et al., 2016). In the preliminary study, it is noteworthy that when exogenously supplied with the bFGF, the CD81 and nestin double-positive dental pulp cells localized in the apical portion were deemed to be mainly responsible for the neural regeneration (Sasaki et al., 2008).

In summary, the results from the aforementioned studies emphasize the important role of the bFGF in neural differentiation and promise new therapeutic strategies by using the bFGF to treat neurological diseases and repair neuronal damage, such as spinal cord injury (SCI), Parkinson’s disease, neonatal hypoxia-ischemia (NHI), and Alzheimer’s disease (Barzilay et al., 2008; Zhang et al., 2014; Luo et al., 2018a; Ye et al., 2018). However, achieving the neurite growth in root canals with the treatment of the bFGF needs more experimental reports.

### Vasculogenesis/Angiogenesis

Angiogenesis is important for tissue regeneration, especially for the dental pulp, since the nutrition of dental pulp could only be provided from the apical foramen of the tooth. The greatest challenge of tissue engineering the “pulp” is to achieve *in vivo* revascularization from the host blood supply (Wigler et al., 2013). The angiogenesis process refers to the migration, proliferation, and differentiation (tube formation) of vascular endothelial cells and is mediated by various angiogenic factors acting on endothelial cells and pericytes (mural cells) (Chung and Ferrara, 2011).

The bFGF was generally described as a protective factor that protects endothelial cells from the programmed cell death process (Karsan et al., 1997; Takeuchi et al., 2015) and was also recognized as one of the potent angiogenesis inducers that prompt vascular formation by stimulating the migration, proliferation, and differentiation of endothelial cells in a dose- and time-dependent manner (Kanda et al., 1999; Kitamura et al., 2014; Zbinden et al., 2018). The angiogenic effect of the bFGF was evaluated similar to that of the VEGF when at the same dosage (Mullane et al., 2008).

DPSCs were shown to locate adjacent to vascular tubes assuming a pericyte location and have also been demonstrated to display potential pericyte-like topography and function. It was reported that the majority of DPSCs expressed the typical pericyte markers including alpha-smooth muscle actin (α-SMA), NG2, PDGFRβ, CD146, and 3G5 (Nam et al., 2017). The pericyte-like properties of DPSCs were investigated to enhance angiogenesis by stabilizing the preexisting vessel-like structures formed by endothelial cells and increasing their longevity, leading to more mature tube-like structures’ formation *in vitro via* the EphrinB2/EphB4 signaling pathway (Dissanayaka et al., 2012; Janebodin et al., 2013; Gong et al., 2019). Meanwhile, the bFGF treatment was able to augment the expression of NG2 and α-SMA of DPSCs and finally, supported the stabilization of HUVEC tubes for a longer time (Delle et al., 2019). In addition, bFGF priming had a stronger impact on DPSCs than hypoxia and enhanced the proangiogenic effect of DPSCs through the secretion of the HGF (hepatocyte growth factor) and the VEGF (Gorin et al., 2016).

In conclusion, the bFGF is an angiogenic factor targeting endothelial cells directly and is capable of facilitating angiogenesis by enhancing the pericyte-like properties of DPSCs.

### Cytokine Production, Matrix Turnover, and Anti-Apoptosis

Beyond that, available studies replenished the bFGF with the abilities of cytokine production, matrix turnover, and anti-apoptosis.

It was stated that the bFGF enhanced dental pulp repair/regeneration by upregulating the expression of several cytokines, including IL-6, IL-8, MCP-1, MIP-1α, and CCL20, *via* the PKC/PI3K-AKT/MAPK signaling pathways. The bFGF-induced cytokines could trigger the innate cellular responses in dental pulp to promote cell differentiation and dentin formation (Kim Y.-S. et al., 2010).

The bFGF may also influence the extracellular matrix turnover through stimulating TIMP-1 expression in HDPCs and SCAPs (Chang et al., 2017; Chang et al., 2020) while inhibiting the expression of type I collagen and downregulating related genes in SHEDs (COL5A1, COL8A2, COL11A1, and COL15A1) and periodontal ligament stem/progenitor cells (COL1A1, COL3A1, ACTA2, and FBN1) (Nowwarote et al., 2020). The influence of the bFGF on collagen genes raised a hypothesis that the bFGF may act as an anti-fibrotic agent in several cell types (Grella et al., 2016). How the bFGF affects pulp repair and regeneration through matrix turnover awaits further investigation.

Besides, the bFGF was reputed to be a survival factor which inhibited cellular senescence and apoptosis in adipose-derived mesenchymal stromal cells (ASCs), probably by diminishing the expression of SA-βgal, p21, and p53 (Nawrocka et al., 2017). A similar anti-apoptosis impact of the bFGF was also underscored when being applied to neural precursor cells (NPCs) (Mellisa and Kristi, 2006), indicating that the bFGF is a potential therapeutic agent in stem cell–based tissue regeneration, while the anti-senescence and anti-apoptosis properties of the bFGF in dental stem cells need more elaboration.

## The Potential Therapeutic Strategies of Basic Fibroblast Growth Factor in Pulp Repair and Regeneration

The bFGF is certified for safe usage and has already been applied in the treatment of ulcer and burns (Akita et al., 2008). The bFGF has also contributed much to the field of nerve (Matsumine et al., 2016), muscle (Goto et al., 2020), and bone regeneration (Chen et al., 2011) based on its prominent regenerative potential. As mentioned above, the bFGF has combinatorial trophic effects on dental stem cells for pulp repair/regeneration to either induce cell migration, proliferation, and differentiation or regulate matrix turnover, cytokine production, and the apoptosis procedure. Therefore, the bFGF can be confirmed to be a safe and competent candidate for clinical applications in pulp regeneration therapy. To sustain this view, we collect the therapeutic protocols which have been developed to apply the bFGF in pulp repair and regeneration therapy.

The regenerative endodontic treatment (RET) contains two different clinical approaches. The first aims to achieve local pulpal regeneration and dentin bridge formation *via* pulp-capping agent usage to stimulate the healing process and preserve the pulp vitality (referred to as “pulp repair”). Another approach strives to induce the growth of new pulp-like tissue in the entire disinfected root canal space in order to revitalize the teeth (referred to as “pulp regeneration”) (Orti et al., 2018).

### Pulp Repair

When the dental pulp is exposed to external stimuli such as caries infection or traumatic injuries, the preservation of dental pulp or the dentin-pulp complex and the maintenance of its viability are essential to avoid serious consequences such as tooth extraction (Kitamura et al., 2012). The formation of new dentin in the site of dentin defects is essential to the local regeneration of the dentin-pulp complex (Kitamura et al., 2012).

**FIGURE 3:**
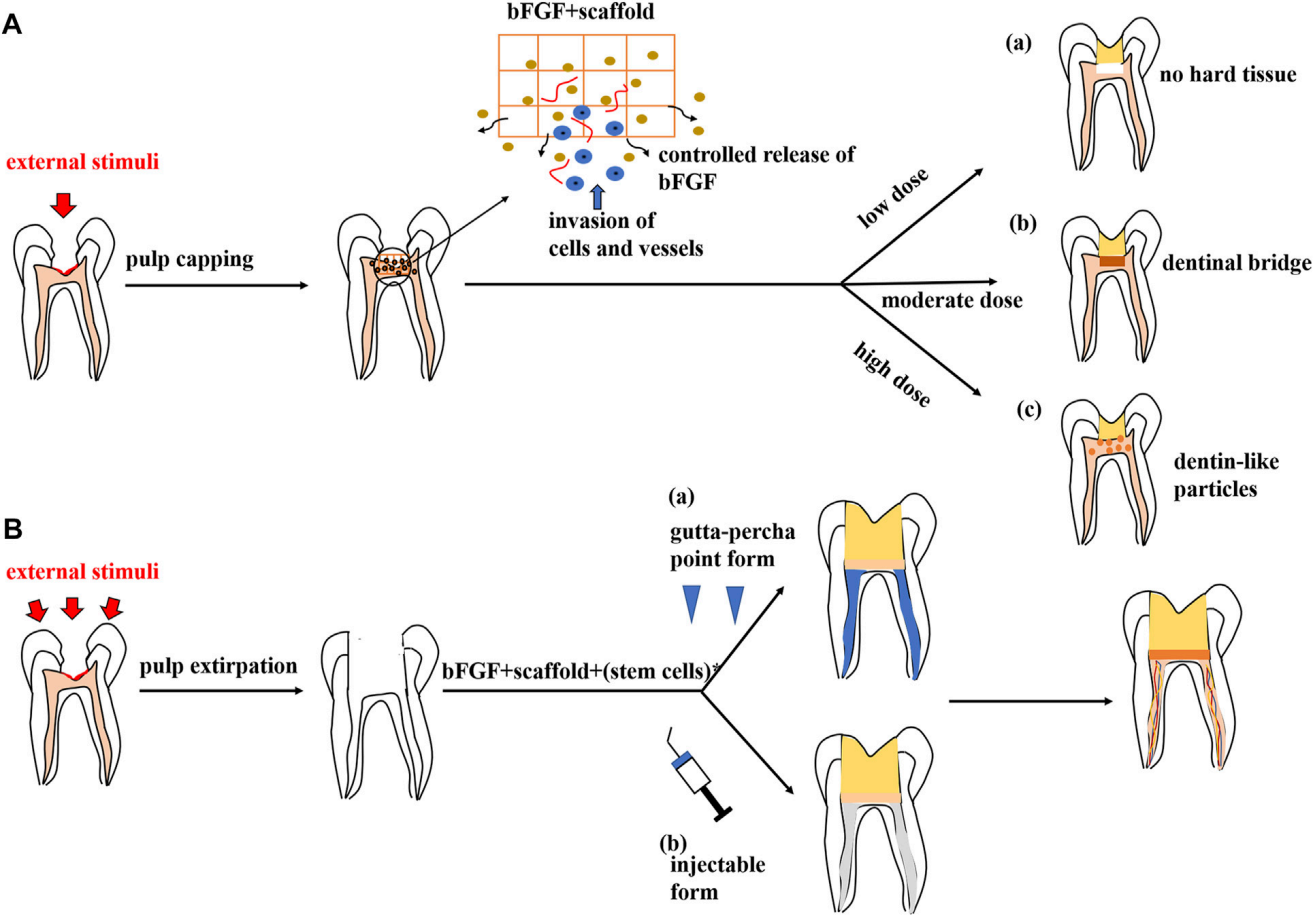
Potential therapeutic strategies of the bFGF in regenerative endodontic therapy. **(A)** Pulp repair. The bFGF could be prepared as a pulp-capping agent when combined with a customized scaffold. This drug carrier system could provide controlled release of the bFGF and induce the ingrowth of cells and vessels, resulting in local pulp regeneration and dentine formation. The outcomes are dose-dependent: (a) low dose of the bFGF (0,0.05 mg/ml), no calcified tissue formation; (b) moderate dose of the bFGF (0.5 mg/ml), dentinal bridge–like structure on the dentin defect surface; and (c) high dose of the bFGF (1.0, 5.0 mg/ml), dentin-like particles in the proliferating pulp. **(B)** Pulp regeneration. A specific scaffold in the gutta-percha point or an injectable form loads the bFGF or the combinatory group of factors, with or without stem cells, which could be applied in pulp regeneration therapy, leading to pulp-like tissue formation in root canals.

For the pulp exposures, at the reversible stage, pulpal inflammation is treated using a direct pulp-capping technique. Calcium hydroxide and mineral trioxide aggregate (MTA) are widely used as pulp-capping materials due to their properties of mobilizing extracellular molecules which then initiate specific biological actions and promote dentin bridge formation (Graham et al., 2006; Li et al., 2015; Tomson et al., 2017; Torabinejad et al., 2018). However, the materials above have been criticized for their limited capacity in tissue regeneration (Chakka et al., 2020) and the tunnel defects in the formed dentin bridge (Chen et al., 2017). Moreover, the treatments elicit the formation of a thin layer of necrotic tissue underneath the capping material (de Souza Costa et al., 2008). Therefore, it is clear that in the future, pulp-capping procedures will rely on a more biological approach (Chakka et al., 2020). Promisingly, bFGF-loaded MesoCS nanoparticles have been shown to be capable of upregulating the odontogenic-related protein of hDPCs (Huang et al., 2017). Following studies have correspondingly reported the local applications of the bFGF as a bioactive molecule that received promising results under the pulp exposure condition. Research has succeeded in treating the pulp exposure through combining the bFGF with the HAP nanoparticle–assembled powder (nano-HAP) and applying this compound to the exposed dental pulp of rat molars. Histological and radiological results showed that the application of the nano-HAP/bFGF induced the invasion of dental pulp cells and vessels, indicating the regeneration of pulp tissue, and stimulated the formation of a dentinal bridge containing numerous dentinal tubules (Imura et al., 2019). It was also validated that the appropriate dosage of the bFGF releasing from gelatin hydrogels could induce the formation of the dentinal bridge-like osteodentin on the surface of the regenerated pulp (Kitamura et al., 2012). The result with this perspective was in accordance with the report provided by Ishimatsu et al., who found that the structures of newly formed calcified tissue in the dentin defect were dependent on the dose of the bFGF incorporated in gelatin hydrogels (Ishimatsu et al., 2009). As far as the studies demonstrated, a high dose (1.0, 5.0 mg/ml) of the bFGF induced calcified particles in the proliferating pulp, which was consistent with the previous results (Kikuchi et al., 2007), whereas a moderate dose (0.5 mg/ml) of the bFGF induced a dentinal bridge–like structure on the surface of the dentin defect. Meanwhile, at a low dose (0, 0.05 mg/ml) of the bFGF, there was no calcified tissue formation (Ishimatsu et al., 2009) (as shown in Figure 3A).

Intriguingly, we find that all experimental models depicted above have used specific scaffolds as drug carriers to load the bFGF, including MesoCS nanoparticles, nano-HAP powder, and gelatin hydrogels, respectively. More scaffold materials can be established for pulp engineering, such as extracellular matrices, self-assembling peptides, and bio-ceramics (Galler et al., 2011). Research studies have shown that scaffold usage could provide sustained (controlled) release of the bFGF, avoiding a large usage of the bFGF protein because of its rapid release (Huang et al., 2017). Furthermore, the comparison of the free-bFGF with the loaded-bFGF was made by putting them into dentin defects above amputated pulp, and it was found that a noncontrolled release of the bFGF only accelerated reparative dentin formation in the residual dental pulp, whereas a controlled release of the bFGF from specific scaffolds induced the formation of DMP-1–positive and nestin-negative osteodentin in the pulp proliferating at the dentin defects, which indicated dentin regeneration (Ishimatsu et al., 2009; Kitamura et al., 2012).

As alluded to above, the application of a moderate dosage (0.5 mg/ml) of the bFGF with a specific scaffold as a drug carrier could induce local pulp regeneration and dentin bridge–like osteodentin formation, which is distinct from the conventional calcium hydroxide or mineral trioxide aggregate treatments. Considering the cost-effectiveness and the short half-life of the bFGF protein, a more current and efficient approach has been proposed to utilize gene-activated scaffolds for dental pulp capping. Chakka et al. transfected DPSCs with nanoplexes comprising polyethyleneimine (PEI) and plasmid DNA (pDNA) encoding for the bFGF and/or BMP-2. Loaded with collagen scaffolds, these nanoplexes resulted in increased expression of the bFGF and/or BMP-2 and enhanced the proliferation and mineralization properties of DPSCs (Chakka et al., 2020). This biological gene delivery approach in pulp capping is expected to facilitate a better pulp regeneration *in vivo* (Chakka et al., 2020).

### Pulp Regeneration

When the external stimuli cause irreversible damage to dental pulp, the affected teeth can be traditionally treated with root canal therapy (RCT), which controls the infection while depriving the teeth of sensitivity and decreasing the resistance of the teeth because of pulp extirpation and malnutrition (Sjögren et al., 1990). In this situation, the pulp regeneration strategy is needed to regenerate a new vital tissue, which ideally mimics the dental pulp in order to extend the longevity of the teeth and improve the patients’ life quality (Simon et al., 2011).

Appropriate cells (stem or progenitor cells), customized scaffolds, and appropriate signaling molecules are required for tissue engineering. Accordingly, this concept can be applied to dental pulp regeneration (Orti et al., 2018). Several reports have documented the regeneration of pulp-like tissue *in vitro via* transplantation of dental pulp stem cells with scaffolds incorporating the bFGF. Self-assembling peptide nanofibers were fabricated to encapsulate dental pulp stem cells and growth factors, including the bFGF, TGF-β1, and VEGF. Subcutaneous transplantation of the component within dentin cylinders into immunocompromised mice showed the formation of a vascularized soft connective tissue similar to dental pulp (Galler et al., 2012). In another approach, the combination of the bFGF and BMP-4 was mixed with DPSCs in a collagen scaffold and was transplanted into a special tissue-engineering chamber. Newly formed tissue with blood vessel formation and DSPP-positive matrix production was later observed in this chamber (Srisuwan et al., 2012). A similar result was acquired in an ectopic root canal transplantation model in which the bFGF and DPSCs were packaged in a silk fibroin scaffold (Yang et al., 2015). It is also noteworthy that even without stem cell transplantation, the bFGF alone or with a combinatory group of growth factors could induce pulp regeneration due to their chemotactic properties. For example, the bFGF was shown to be able to yield recellularization and revascularization in endodontically treated human teeth which were implanted into the dorsum of rats (Suzuki et al., 2011). Meanwhile, the bFGF combined with a basal set of growth factors (NGF and BMP-7) also induced the regeneration of pulp-like tissue in the entire root canal from the root apex to the pulp chamber (Kim J. Y. et al., 2010). Thus, it is plausible to postulate a cell-free therapy which works by using selective chemotactic cytokines to recruit stem cells from residual pulp or from the periapical region and subsequently induce the regeneration of dental pulp. If this concept proved to be successful, cell-free therapy may be an advantageous approach that means a more reliable, feasible, and affordable alternative to the cell transplantation method (Galler et al., 2014). It is also thought-provoking that the bFGF was usually applied with a combination of other growth factors in the pulp regeneration therapy. These growth factors (like VEGF, BMP-2, and TGF-β1) were said to have positive effects on pulp regeneration or proved to have a synergistic effect with the bFGF to enhance specific biological effects (He et al., 2008; Kang W. et al., 2019; Zhang et al., 2020). Other than the reciprocal growth factors, the supplementation of inorganic polyphosphate [poly(P)] was confirmed to facilitate the autocrine of the bFGF and enhance the function of the bFGF by promoting its stability and receptor affinity, resulting in the enhancement of the proliferation of HDPCs (Kawazoe et al., 2008). Intraflagellar transport protein 80 (IFT80) (Yuan et al., 2019b) and FGF-binding protein 1 (FGF-BP1) (Tassi et al., 2001) were also proposed as positive modulators in bFGF signal transduction. Therefore, future studies need to be carried out to understand the interplayed relationships among these key players and develop an optimal mixture of molecules for pulp regeneration (Schmalz et al., 2017). Besides, ongoing work is also required to determine whether the bFGF, a single cytokine, is sufficient for the regeneration of dental pulp (Kim J. Y. et al., 2010). For clinical concerns, Bhoj et al. took a first step to fabricate an RGD-bearing alginate scaffold that replicated the shape of gutta-percha. With the encapsulation of DPSCs and dual growth factors involving the bFGF and the VEGF, the customized RGD-bearing alginate framework could be simply shaped to fill the pulp space (Bhoj et al., 2015). In terms of future clinical application, scaffold materials could also be developed into an injectable form to achieve a more efficient root canal obtruration (Marler et al., 2000) (as shown in Figure 3B).

Collectively, these reports support that the bFGF is a validated candidate for future treatment in pulp regeneration.

## Summary

To sum up, the bFGF is known to be involved in all stages of pulp repair/regeneration and uses different intracellular signaling pathways to control specific biological processes, which may be determined by cell origins and treatment durations (Lee et al., 2015; Novais et al., 2019; Nowwarote et al., 2020). Abundant molecules and proteins are involved in such processes. In terms of clinical use, a moderate dose of the bFGF combined with a customized scaffold could be prepared as pulp-capping agents to preserve the vitality of the dentine-pulp complex. Furthermore, the bFGF combined with stem cells and specific scaffolds is put into use in pulp regeneration therapy. In the near future, it is more advisable to begin a new paradigm in pulp regeneration with the cell-free approach (without exogeneous cell transplantation), cytokine reduction strategy (using only one or a minimum subset of cytokines), and injectable scaffold form (Kim J. Y. et al., 2010; Galler et al., 2011; Galler et al., 2014). At last, since the bFGF has attracted much attention in regenerative endodontic therapy, it is a wise choice to focus on its siblings. Not surprisingly, other FGFs, like FGF-8 (Tsikandelova et al., 2018), FGF-9 (Dai et al., 2012), and aFGF in particular (Cam et al., 1992; Li and Sae-Lim, 2007), are confirmed to own non-negligible regenerative potential in dentistry and tissue engineering. Therefore, greater effort should be put forward to exploit more regenerative properties of FGFs to reinforce their role in pulp repair and regeneration.
